# Numerical Simulation of the Effect of Electrical Stimulation on Disuse After Hip Replacement

**DOI:** 10.3390/biomedicines13020471

**Published:** 2025-02-14

**Authors:** Qian Wang, Chuanyong Qu, Xiaohui Li, Yufan Yan

**Affiliations:** 1 Department of Mechanics, School of Mechanical Engineering, Tianjin University, Tianjin 300350, China; qianofwang@tiu.edu.cn (Q.W.); yan_yufan@tju.edu.cn (Y.Y.); 2 Department of Joint Surgery, Tianjin Hospital, Tianjin University, Tianjin 300211, China

**Keywords:** bone remodeling, total hip replacement (THA), stress shielding, disuse, electrical stimulation

## Abstract

**Background:** Total hip replacement replaces the femoral head, which cannot heal, with an artificial femoral shaft to ensure the patient’s normal life. However, due to the stress-masking effect of the proximal femur loaded with the artificial femur stem, the implant bears a large part of the load, resulting in insufficient stress stimulation of the proximal femur and bone waste remodeling. In turn, it is easy to lose bone, resulting in loosening. As a new treatment method, electrical stimulation has been widely used for bone loss, nonunion, and other diseases, and it has achieved good therapeutic effects. **Methods:** Therefore, in this work, electrical stimulation was introduced for postoperative density assessment, and a new disuse remodeling model was established to simulate density loss after remodeling and the resistance effect of electrical stimulation. The effects of various parameters on density loss in the model are discussed. **Results:** The simulation results revealed significant stress masking and density loss in the neck of the femur after hip replacement, and electrical stimulation placed in the neck of the femur may resist this density loss to a certain extent. The rate of bone mineral density reduction decreased after the addition of electrical stimulation, indicating that electrical stimulation can have a certain resistance to the density reduction caused by stress shielding, and this result is helpful for the rehabilitation of hip arthroplasty.

## 1. Introduction

The hip joint is composed of the femoral head and acetabulum; as one of the joints supporting human movement, it is easily affected by impact loading or fatigue loading, resulting in structural failure. Fracture of the neck of the femur can cause serious damage to the human body, especially for elderly individuals with weak recovery ability. THA has been used as a treatment for femoral neck fracture for many years and has achieved remarkable results [1]. The implant can cause structural changes in the human femur, leading to a stress-shielding effect, which reduces mechanical stimulation in some areas of bone tissue and leads to bone disuse remodeling [2]. A decrease in bone tissue density can cause significant stress shielding at the proximal femur, which can lead to bone remodeling, bone transport, and, in some cases, early loosening [3], as well as the need for re-revision surgery [4]. Therefore, it is necessary to consider how to reduce the stress-shielding effect caused by implant implantation.

The stress-shielding effect is one reason why hip replacement surgeries fail. Generally, the decrease in bone density around the prosthesis caused by stress shielding depends on the characteristics of the implant, such as the material or the implant method. Mechanics-based models can be used to predict bone fitness, and they rely on mechanical stimuli such as stress, strain, and strain energy [5]. In recent years, many studies have focused on reducing the effects of stress shielding, developing a common method to design implants with different structures and comparing and analyzing the stress-shielding effect via the finite element method to improve the implant structure and implantation method [6,7]. Das and Chakraborti [8] and Mihalko et al. [9] analyzed the impacts of new biomaterial implants on hip arthroplasty. New biomaterials can improve the risk of wear infection and other problems to a certain extent and have great development prospects. Both the material and implant characteristics are designed to reduce the stress-shielding effect.

Early experimental studies explored the relationship between mechanical load and bone mass. With the advent of the theory of piezoelectricity in bone [10], the influence of electromagnetic signals on bone remodeling has gradually been recognized. On this basis, some scholars have focused on the influence of external stimuli such as electromagnetic fields on bone remodeling through experimental means. Rubin et al. [11] explored the stimulatory effect of electrical stimulation on osteoclasts through in vitro cell experiments, and the experimental results revealed that a sinusoidal electric field at a certain intensity inhibited the formation of osteoclasts. Tschon et al. [12] reported that a pulsed electromagnetic field at a certain intensity and frequency could affect osteoclast development. Hartig et al. [13] studied the effect of a pulsed electric field on the proliferation of osteoblast cells in vitro and noted that an electric field could promote the synthesis of the bone matrix. Clark et al. [14] discussed various parameters of the capacitive electric field and determined the optimal expression state of genes in human bone cells in the electric field. Wiesmann et al. [15] discussed and analyzed the structure and morphology of crystals formed by osteoblasts under the influence of electrical stimulation and noted that electrical stimulation can promote the formation ability of cells. Wang et al. [16] studied the life process of osteoblasts under electrical stimulation; they reported that electrical stimulation of 100 μA/cm^2^ can promote the proliferation of osteoblasts and that electrical stimulation plays a key role in bone metabolism. Yao et al. [17] investigated the dual effects of a tricalcium phosphate-based gelatin scaffold in combination with electroacupuncture stimulation on the activation of osteoclasts and osteoblasts, as well as new bone regrowth in vitro and in vivo, and they showed that electroacupuncture stimulation can enhance osteogenesis and new bone regrowth in vivo. Luo et al. [18] reviewed the knowledge and progress of electrical stimulation for bone repair and expounded the cellular behavior and potential electrical stimulation mechanism of electroactive biomaterials in bone healing, providing new insights into the mechanism of bone regeneration through the use of electroactive biomaterials.

Both mechanical stimulation and electrical stimulation can affect bone remodeling, and bone remodeling under multiple physical fields has also been a hot topic in recent years. The current mainstream theory is that the cellular pathways regulated by growth factors in bone can activate the dynamic expression of osteogenic and osteoclast cells and then indirectly regulate the process of bone remodeling [19]. Although external stimuli such as electric and magnetic fields have been shown to have a significant effect on bone remodeling [20], their internal mechanism still needs to be further explored and discovered. There have been several studies on the prediction of bone density loss via a remodeling model, but few simulation studies have investigated the coupling effect of mechanical stimulation and electrical stimulation on bone mass loss. In this work, the waste of the femoral head caused by stress shielding was studied, and the waste resistance effect of electrical stimulation was investigated. There are few studies on the effect of electrical stimulation on human bone, and studies on the coupling effect of mechanical stimulation and electric stimulation on bone remodeling is lacking. Based on this study, the effect of electrical stimulation on bone waste remodeling was studied. Considering that the in vivo experiment could not be carried out, this paper established a model before and after hip replacement surgery and carried out finite element analysis, analyzed the stress-shielding phenomenon, verified the resistance of electrical stimulation to bone disuse remodeling, and provided help for treatment after hip replacement.

## 2. Disuse Remodeling Model

### 2.1. Finite Element Model, Boundary Conditions, and Material Assignment

**Figure 1:**
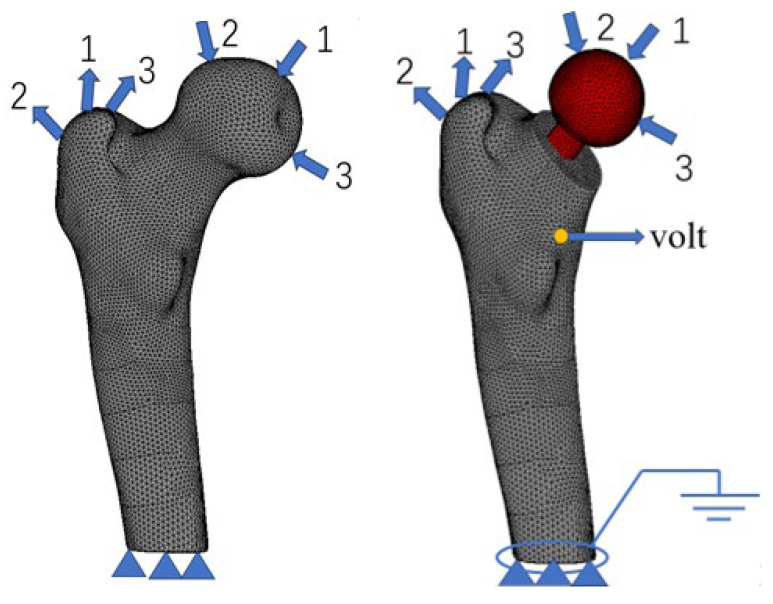
Finite element model of the femur.

In this work, a finite element model of the human proximal femur was established by using DICOM data and reverse-engineering software. The femoral head was intercepted via the Boolean operation, and the artificial femoral shaft and proximal femur were combined. The whole finite element model is divided into 10 tetrahedral node elements. To simulate stress masking after hip replacement, the proximal femur without implantation was used for comparative analysis. The boundary conditions of the two finite element models were the same: fixed constraints were applied at the bottom, and three common working conditions, namely, standing on one leg, hip abduction, and hip adduction, were applied. The specific data are shown in Table 1. The finite element model is shown in Figure 1.

**Figure 2:**
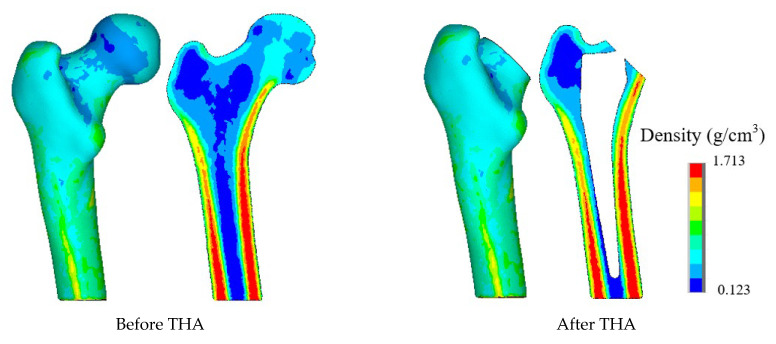
Density distribution.

To better describe the density distribution within the finite element model, in this study, the bone CT values were used to establish a functional relationship with the bone density and CT data of HU as the unit of measurement. In view of the findings of this study, the waste with the remodeling of the proximal femur model, cancellous bone, is an important object of study; thus, Formula (1) [22] was used to establish the relationship between the density and the bone CT value:

(1) $$\rho_{i}=\rho_{a}+\frac{\rho_{b}-\rho_{a}}{HU_{max}-HU_{min}}\left(HU_{i}-HU_{min}\right),$$

where $\rho_{a}$ and $\rho_{b}$ are set to the minimum and maximum density values, respectively; $HU_{max}$ and $HU_{min}$ are the maximum and minimum CT values of the model; $HU_{i}$ is the density value of each unit; the material gradient is set to 50; and Poisson’s ratio is 0.3. The proximal femur model after the completion of the assignment is shown in Figure 2. The bone marrow cavity has the lowest density, and the cancellous bone and bone cortex are also clearly visible.

The determination of the relationship between the elastic modulus and density follows a previous research method. Different elastic moduli and densities are set inside different elements of the whole finite element model, and Poisson’s ratio is uniformly set to 0.3. $E_{t}$ is the modulus of elasticity, and $\rho_{t}$ is the bone density. The relationship between $E_{t}$ and $\rho_{t}$ is shown in Formula (2) [23]:

(2) $$E_{t}=3790\times \rho_{t}^{3},$$

According to Formula (3) [24], the relationship between density and porosity is established:

(3) $$\rho_{t}=\left(1-P_{t}\right)\rho_{0},$$

where $\rho_{0}$ represents the maximum bone density, and $P_{t}$ represents the bone porosity over time.

### 2.2. Bone Disuse Remodeling Model

**Figure 3:**
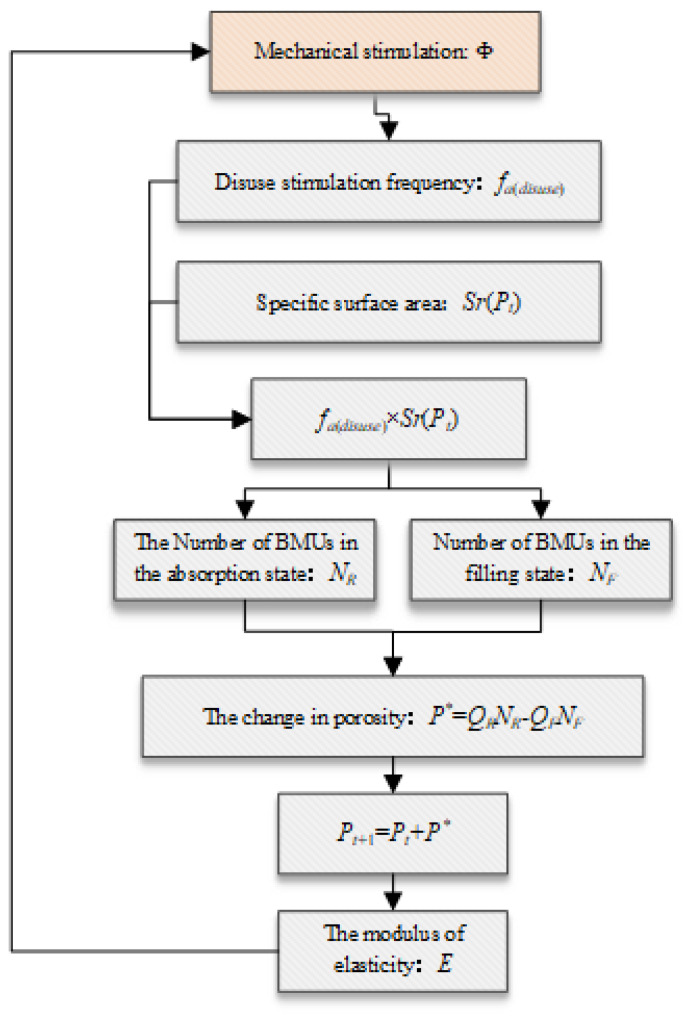
Logical block diagram of the bone-remodeling algorithm.

The whole process of bone waste remodeling is shown in Figure 3 below. Both mechanical stimulation and electrical stimulation can affect the dynamic expression of the activation frequency of bone waste. The activation frequency of bone waste follows previous research results and will be described in detail in the following section. The product of the disuse activation frequency and body surface area was used to describe the BMU quantity per unit volume [25], and the dynamic change in porosity was used to describe the change in bone volume [25]. The model algorithm is described in detail below.

The remodeling model for whole bone disuse has been expanded and extended on the basis of previous methods. The mechanical load is as follows [26]:

(4) $$\Phi ={\left(\sum_{i=1}^{N}n_{j}{{\bar{\sigma }}_{j}}^{m}\right)}^{1/m},$$

where *N* is the total number of loading conditions, which is set to 3 in this study. $n_{j}$ is the frequency of load stimulation under each condition. ${\bar{\sigma }}_{j}$ is the equivalent stress under a single working condition, and *m* is the regulating factor, with a value of 4.

(5) $$f_{a(disuse)}=\frac{f_{amax}}{1+e^{\left(\Phi /\Phi_{0}-\kappa_{D1}\right)/\kappa_{D2}}},$$

where $f_{amax}$ is the maximum waste frequency, $f_{a(disuse)}$ is the daily waste frequency, Φ is the daily load stimulus, and $\Phi_{0}$ is the daily load stimulus under the equilibrium state. The calculation formula is shown in Formula (5) [25]. See [27] for the calculation formula of $\bar{\sigma }$, where the unit is MPa, and the equivalent stress is calculated via the following formula. $\kappa_{D1}$ and $\kappa_{D2}$ are shape control factors; please refer to Table 2 for the specific values.

(6) $$\Phi_{0}=(6000\times {\bar{\sigma }}_{1}^{m}+2000\times {\bar{\sigma }}_{2}^{m}+2000\times {\bar{\sigma }}_{3}^{m}{)}^{1/m},$$

(7) $$\bar{\sigma }=\sqrt{\frac{1}{2}[{\left(\sigma_{1}-\sigma_{2}\right)}^{2}+{\left(\sigma_{2}-\sigma_{3}\right)}^{2}+{\left(\sigma_{3}-\sigma_{1}\right)}^{2}]},$$

(8) $$S_{r}\left(P_{t}\right)=32.1\times P_{t}-93.9\times P_{t}^{2}+134\times P_{t}^{3}-101\times P_{t}^{4}+28.8\times P_{t}^{5},$$

Bone, as a representative biological material of the human body, can be divided into two main categories. For both cortical bone and cancellous bone, there are a certain number of pores in the interior that expand the specific surface area. In this work, the results of previous studies were as follows (see Formula (8)) [30], and the porosity was used to describe the dynamic change in the specific surface area. $S_{r}\left(P_{t}\right)$ is the specific surface area per unit volume of bone tissue, which is a polynomial function related to porosity $P_{t}$ (see Formula (8)). Bone remodeling begins on the internal surface of the tissue, so the product of $S_{r}\left(P_{t}\right)$ and the disuse activation frequency $f_{a}$ represents BMU activation per unit volume.

(9) $$N_{R}=\int_{t-T_{R}}^{t}f_{a(disuse)}S_{r}(P_{t})dt,$$

(10) $$N_{F}=\int_{t-T_{R}-T_{I}-T_{F}}^{t-T_{R}-T_{I}}f_{a(disuse)}S_{r}(P_{t})dt,$$

The product of the specific surface area and activation frequency of waste was used to describe the effect of BMU on bone tissue caused by waste stimulation. Hazelwood’s model was used in the whole integration process, and *N_R_* and *N_F_* were the BMU numbers in the absorption state and filling state, respectively. *T_R_*, *T_I_*, and *T_F_* represent the absorption, reversal, and filling stages, respectively, of the BMU. The calculation formulas for specific values are Formula (9) [25] and Formula (10) [25], as shown in Table 2.

(11) $$P^{*}=Q_{R}N_{R}-Q_{F}N_{F},$$

**Figure 4:**
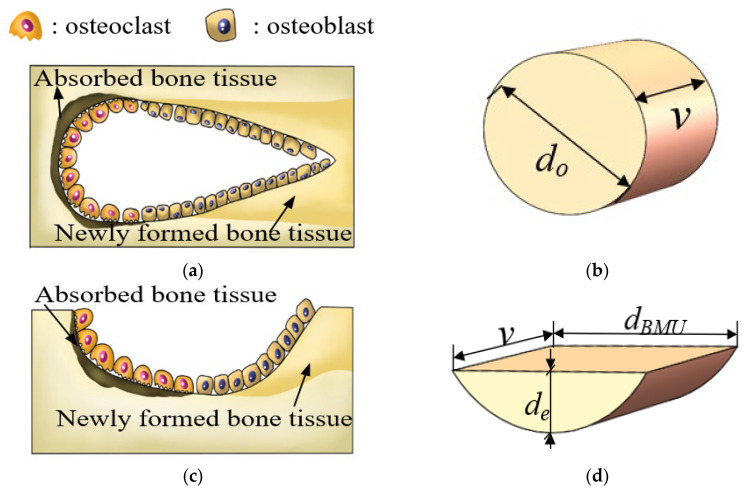
Primitive and simplified models of BMU in bone cortex and cancellous bone: (**a**) the primitive model of BMU in cortical bone; (**b**) the simplified model of BMU in cortical bone; (**c**) the primitive model of BMU in cancellous bone; (**d**) the simplified model of BMU in cancellous bone.

The absorption and filling process of the BMU regulates the change in porosity, as shown in Formula (11) [13] above, where *Q_R_* and *Q_F_* are the absorption rate and filling rate of the BMU, respectively, as defined in Formula (12) [25] and Formula (13) [25]. Owing to the different internal morphologies of BMU in the bone cortex and cancellous bone, the two BMU models are simplified in this chapter. As shown in Figure 4, the osteoclast population in the BMU in the bone cortex first absorbs bone tissue, then the osteoblast population fills the extracted cavity on this basis, and the whole BMU absorbs and fills the bone tissue in a cylindrical shape. In the interior of spongy bone, osteoclasts act on the surface of the bone trabeculae, absorbing surface bone tissue in the shape of grooves, thus simplifying its shape into a semi-elliptic cylindrical shape. See Table 2 for the geometric parameters. v is the daily travel distance of the BMU; $d_{e}$, $d_{BMU}$, and $d_{o}$ are the structural parameters of the BMU; and the absorption process and filling process of the BMU in the equilibrium state are set to balance each other.

(12) $$Q_{R\_can\;}=\frac{\pi d_{e}d_{BMU}v}{4},$$

(13) $$Q_{R_{-}cor\;}=\frac{\pi d_{o}^{2}v}{4},$$

## 3. Disuse Remodeling Process of the Proximal Femur

### 3.1. Stress-Shielding Effect

In this section, mechanical loads with the same direction and equal size were applied to the proximal femur model without an artificial hip joint and the model after implantation, and completely consistent fixed displacement boundary conditions were set at the bottom of the femur. After the two statistical analyses, the stress data of the proximal femur under the two working conditions were extracted. The stress value of the bone tissue after implantation was divided by the stress value in the working condition without the implant, and the stress shielding of the model was explored through the ratio.

**Figure 5:**
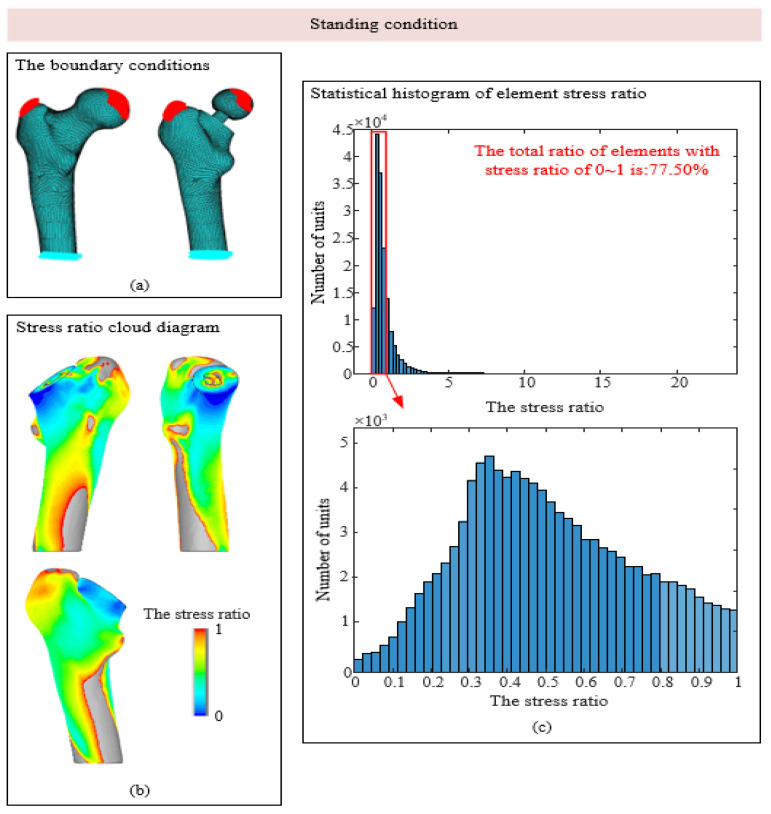
Stress shadowing cloud map and unit statistics under the standing condition. (**a**) The boundary conditions; (**b**) Stress ratio cloud diagram; (**c**) Statistical histogram of element stress ratio.

As shown in Figure 5c, under the condition of standing on one leg, the stress ratio of most units of the femur model after implantation is within 0~2, and the units with stress ratios of 0~1 are in the red box, accounting for 77.5% of the total units, indicating that stress shielding exists to a certain extent in most areas of the model. The histogram with a stress ratio of 0~1 was subdivided. Most of the elements with stress shielding were in the range of 0.2~0.7, and most elements were near 0.4. In the condition of hip adduction, the proportion of elements with stress ratios between 0 and 1 is approximately 43%, which is much smaller than that of 77.5% in the condition of standing on one leg. In the condition of hip abduction, the proportion of elements with stress shielding is approximately 86%, which is the most obvious condition with stress shielding among the three conditions.

**Figure 6:**
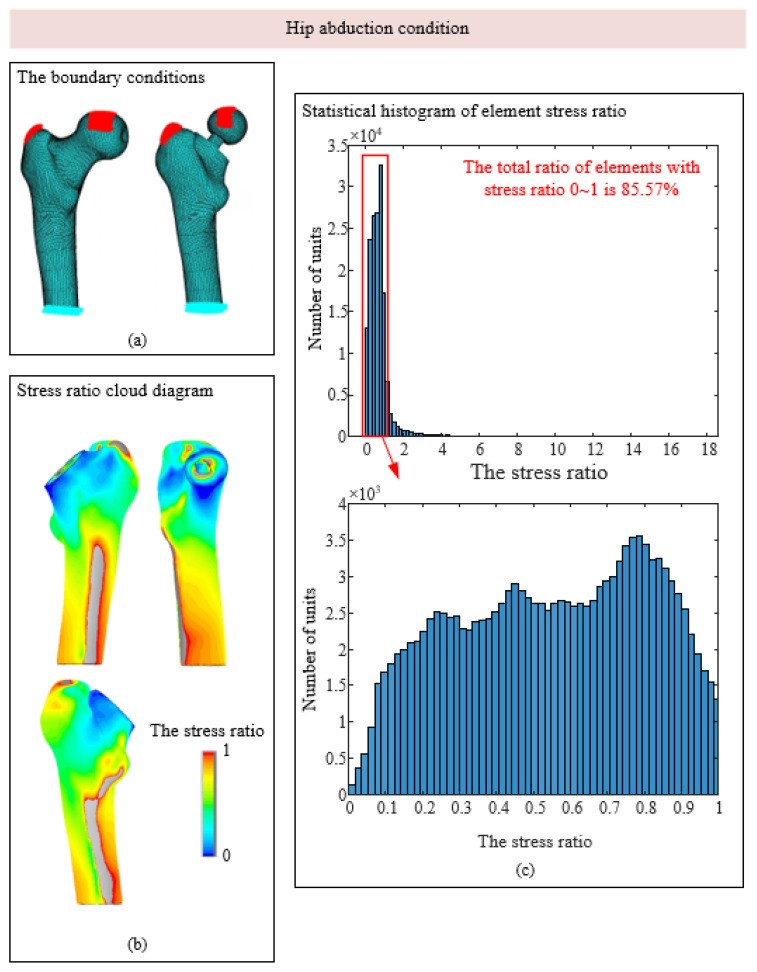
Stress shadowing cloud map and unit statistics under hip abduction conditions. (**a**) The boundary conditions; (**b**) Stress ratio cloud diagram; (**c**) Statistical histogram of element stress ratio.

**Figure 7:**
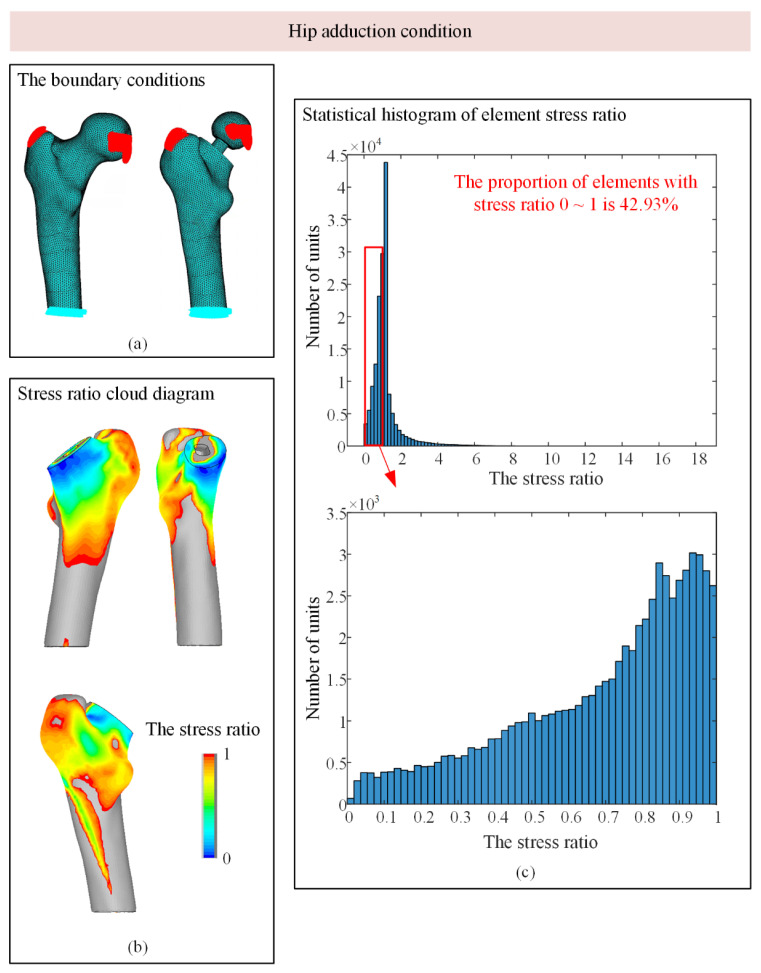
Stress shadowing cloud map and unit statistics under hip adduction conditions. (**a**) The boundary conditions; (**b**) Stress ratio cloud diagram; (**c**) Statistical histogram of element stress ratio.

As shown in Figure 5b, Figure 6b and Figure 7b, the area with stress occlusion generally exists in the neck of the femur under the three working conditions. Normally, the neck of the femur bears a large mechanical burden, but after the artificial hip joint is implanted, the load that should be borne by the neck of the femur is replaced by the prosthesis so that there are many areas of low stress in the neck of the femur. According to Wolff’s Law, when bone tissue is stimulated by a low load for a long time, internal density is lost, and the density loss of the femoral neck significantly reduces its load-bearing capacity, which continuously increases the secondary revision rate. The stress occlusion of femoral cadres is not obvious, and the stress ratio in some areas is even greater than 1. This is because mechanical stimulation is attached to the prosthesis, and the artificial stem of the prosthesis is fitted with the femoral shaft by a mud line; thus, the prosthesis attaches part of the load to the femoral shaft, resulting in increased stress in some areas.

### 3.2. Numerical Simulation of Outages Under Mechanical–Electrical Coupling

In this work, the dielectric tensor of bone material is defined as a diagonal matrix containing two known parameters [31]:

(14) $$\beta =\left[\begin{array}{lll} \beta_{11} & & \\ & \beta_{11} & \\ & & \beta_{33} \end{array}\right],$$

In Formula (14), $\beta_{11}=88.54\times 10^{-12}\;F/m$, and $\beta_{33}=106.248\times 10^{-12}\;F/m$. In this study, taking into account the acceptable range of normal human voltages, a voltage of 32 V was set at the neck of the femur model, and the boundary condition of voltage 0 [32] was set at the bottom of the model. For specific placement, please refer to Figure 1.

**Figure 8:**
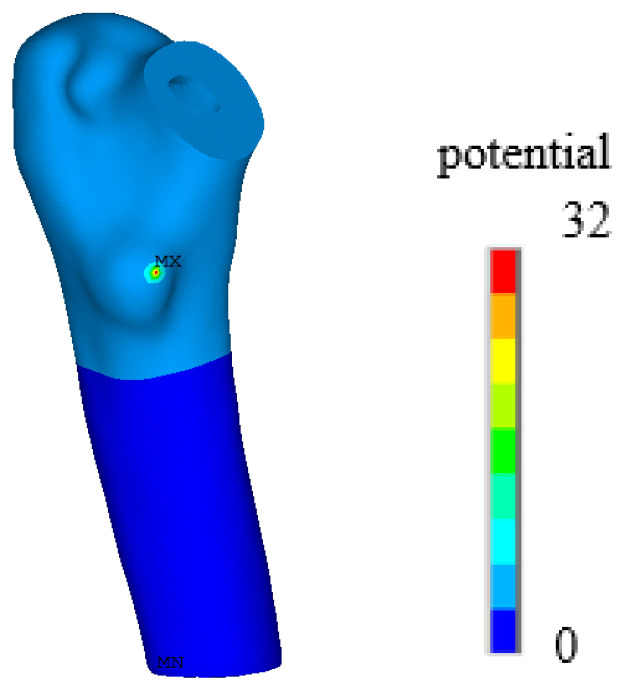
The overall potential distribution after the charge is applied.

**Figure 9:**
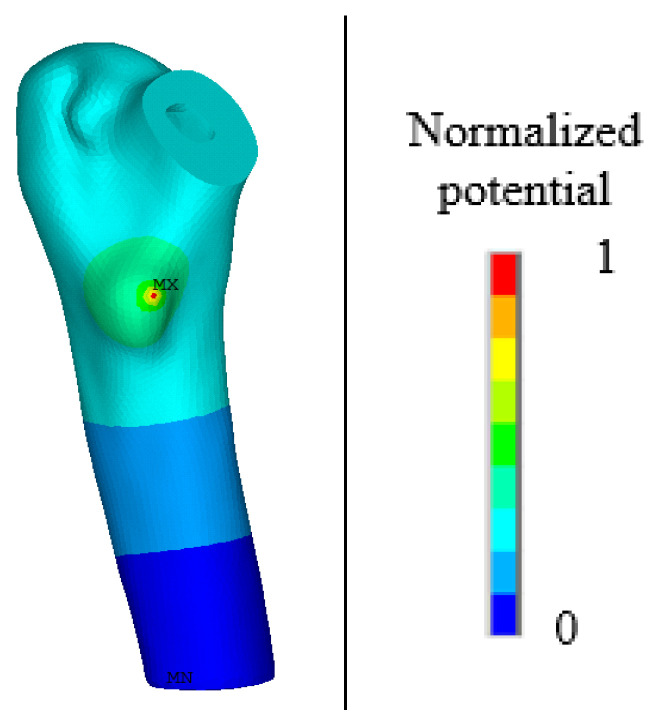
Normalized potential distribution diagram.

To describe the phenomenon of waste resistance to electrical stimulation, this paper proposes a phenomenological model in which the electric field stimulation stage, electric field stimulation, and electric field stimulation activate the BMU frequency to regulate the absorption of osteoclasts and osteoblasts by changing the parameters of the activation frequency to regulate the absorption of osteoclasts and osteoblasts. Four cases, with amplitudes of 0%, 30%, 50%, and 70%, are discussed. The normalized electrical potential was used to measure the effect of electrical stimulation on bone remodeling. The area with the highest potential has the greatest influence and is set to 1, whereas the area with the lowest potential has the least influence and is set to 0. The normalized potential value in each unit is different, and the product of the normalized potential value and activation frequency under different amplitudes can describe the effect of electrical stimulation on the activation frequency of disuse. Electrostatic analysis was performed on the proximal femur model after implantation, and a potential diagram before and after normalization is shown in Figure 8 and Figure 9.

**Figure 10:**
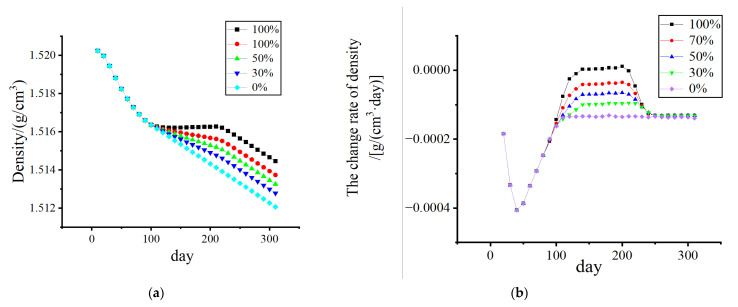
The variation in cortical bone density and the rate of change in density with time. (**a**) Density change; (**b**) The density change rate changes.

**Figure 11:**
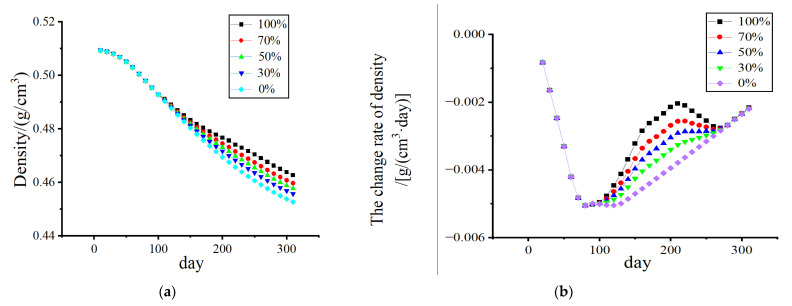
The variation in cancellous bone density and the rate of change in density with time. (**a**) Density change; (**b**) The density change rate changes.

Figure 10 and Figure 11 show that, without electrical stimulation, the density of cortical bone and cancellous bone at the proximal end of the femur decreased significantly within 1–100 days due to the stress-shielding effect. From days 101 to 200, the number of osteoclasts and osteoblasts decreased, and the rate of density change decreased due to the electrical stimulation of the cortex during remodeling. As the amplitude changed from 0% to 70%, the density in the bone cortex transitioned from decreasing to increasing, and the higher the amplitude was, the faster the density increased.

**Figure 12:**
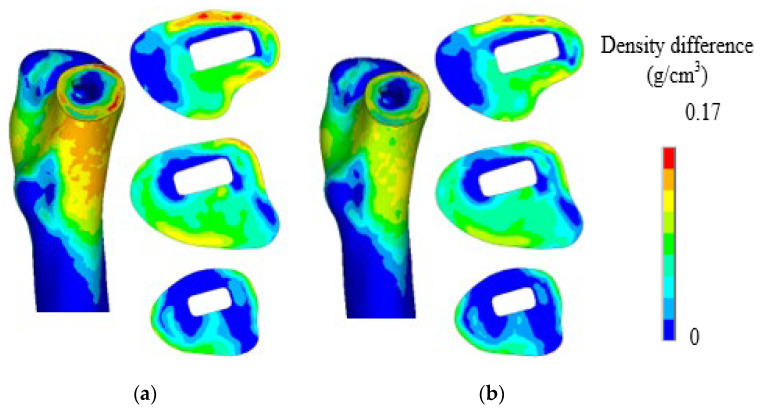
Density difference distribution: (**a**) without electrical stimulation; (**b**) electrically stimulated.

After 300 days of disuse remodeling, the density clouds of nonelectric stimulation and charged stimulation were compared, as shown in Figure 12. Overall, electrical stimulation has a certain inhibitory effect on the density loss of the waste area due to stress shielding and does not affect the density of the element at the normal stress ratio. Without electrical stimulation, the maximum density loss at the neck of the femur was 0.17 g/cm^3^. After receiving electrical stimulation for a certain period of time, the density loss at the neck of the femur gradually decreases with the gradual increase in the influencing factors. To describe the density difference within the femoral neck region, three sections were delineated in this study. Electrical stimulation not only affected density loss outside the femoral neck but also exerted a certain resistance effect inside the femoral neck.

**Figure 13:**
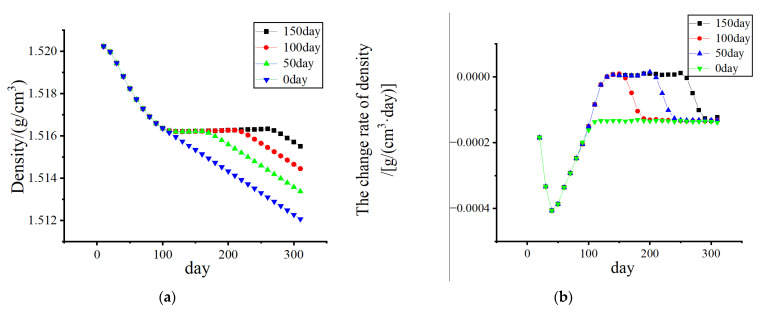
The variation in cortical bone density and the rate of change in density with time. (Add electrical stimulation). (**a**) Density change; (**b**) The density change rate changes.

**Figure 14:**
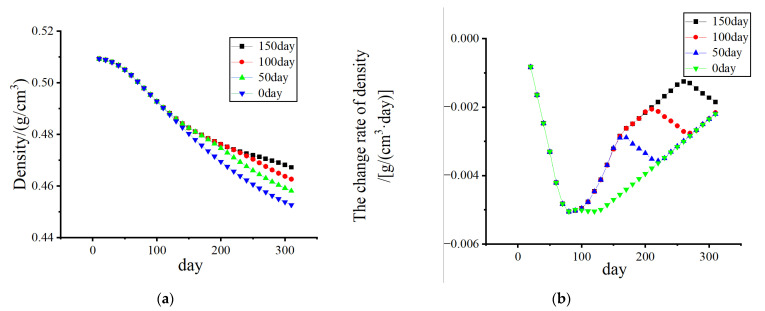
The variation in cancellous bone density and the rate of change in density with time. (Add electrical stimulation). (**a**) Density change; (**b**) The density change rate changes.

Considering the effect of the duration of electrical stimulation, as shown in Figure 13 and Figure 14, the density loss within 300 days was investigated. The starting point of electrical stimulation was the 101st day, and the conditions of 50 days, 100 days, and 150 days were considered. With prolonged electrical stimulation time, the density of cortical bone plateaued, and the change rate of bone density was close to 0 g/(cm^3^·day). When the duration was long enough, the density of cortical bone increased to a certain extent. As shown in Figure 13b, electrical stimulation led to a significant increase in the BMD change rate during the stimulation stage, and once electrical stimulation was stopped, the BMD change rate returned to a negative value. The density loss of cancellous bone was gradually improved with increasing duration of electrical stimulation, and the duration of electrical stimulation was also a key factor affecting bone density.

## 4. Discussion

The hip joint plays a key role in providing dynamic support for the body’s weight and in transferring the load distributed axially from the bone to the lower limbs [33]. Hip joints are prone to a variety of risks and injuries, and in cases of severe damage, hip replacement surgery is needed [34]. After the implant is inserted, the stiffness of the implant is greater than the stiffness of the bone tissue, which causes the load to be transferred from the hip femoral head to the implant [35]; this results in a stress-shielding effect.

In this chapter, a real human femur model is constructed from CT data, and density and elastic modulus distributions close to those of the real model are assigned to those of the bone model. The stress shielding under three representative working conditions and the bone loss caused by stress shielding are discussed through the disuse algorithm. Because the prosthesis bears more load, there is a certain stress shield on the external surface and interior of the neck of the femur after the implantation of the prosthesis [36], which leads to a certain density loss in part of the neck of the femur. Electrical stimulation can promote bone tissue formation [37]. Electric charge stimulation in the neck of the femur can slow this loss trend, indicating that the mechanical–electrical coupling waste utilization algorithm proposed in this paper can be applied to the analysis and study of waste utilization after implantation.

In addition, this study has been simplified in several aspects:

1. In daily life activities, human bones are affected by a variety of loads. This paper discusses three types of typical working conditions: bone tissue with no loadings, bone tissue subjected to ligament forces, and bone subjected to muscle forces. Joint forces, such as the combined impact of a variety of loads, can be studied in future work [38]. A more complex boundary condition of the model will undoubtedly more closely approach the real situation of the simulation results;
2. Bone tissue inside the material is assigned the CT value and density of a functional relationship for determination. In this work, the research on bone tissue, which is an isotropic homogeneous material, refers to the uniform internal porosity distribution in each unit without considering the location of the pore, as a typical anisotropic material [39]. Considering the uniqueness of the internal structure, more realistic simulation results can be obtained;
3. There are also various blood vessels and tissue fluids in real bone tissue, which is essentially a biomaterial composed of solid and liquid [40]; real bone tissue needs to be further explored in future studies. In addition, individual differences in biomaterials must be discussed and analyzed;
4. Electrical stimulation, as an in vitro physical stimulus, exhibits excellence in clinical application; however, at present, the internal mechanism of the impact of the bone-remodeling process still requires further exploration. This phenomenological model can provide a certain explanation for this phenomenon, but a specific parameter model remains to be further modified and perfected according to the experimental data.

## 5. Conclusions

In this work, a disuse remodeling model, including cancellous bone and the bone cortex, was proposed based on previous work. The disuse situation caused by stress occlusion after hip arthroplasty was investigated via this model. On this basis, the influence of mechanical stimulation and electrical stimulation on bone remodeling was also considered in the model. The results show that stress shielding may lead to a certain loss of bone density, and external physical stimulation factors such as electric fields may resist bone density loss to a certain extent. The model proposed in this paper can provide a reference for the clinical guidance of postoperative density prediction and electric field therapy.

## Figures and Tables

**Table 1:** Mechanical loading on the proximal femur [21].

Load Conditions	Cycles	Joint Force		Muscle Force	
		Size (N)	Direction (°)	Size (N)	Direction (°)
1 Standing condition	6000	2317	24	703	28
2 Hip abduction	2000	1158	−15	351	−8
3 Hip adduction	2000	1548	56	468	35

**Table 2:** Parameter value summary.

Constant	Numerical Value	Unit
$\kappa_{D1}$	0.5 [25]	
$\kappa_{D2}$	0.1 [25]	
$f_{amax}$	0.1 [25]	BMUs/(mm^3^·day)
*T_R_* (cortical bone)	24 [25]	day
*T_I_* (cortical bone)	8 [25]	day
*T_F_* (cortical bone)	64 [25]	day
*T_R_* (cancellous bone)	60 [28]	day
*T_I_* (cancellous bone)	57 [28]	day
*T_F_* (cancellous bone)	197 [28]	day
v	0.01 [28]	mm
$d_{BMU}$	0.65 [28]	mm
$d_{e}$	0.05 [28]	mm
$d_{o}$	0.2 [29]	mm

## Data Availability

The raw data supporting the conclusions of this article will be made available by the authors on request.
